# The value of radiomics-based CT combined with machine learning in the diagnosis of occult vertebral fractures

**DOI:** 10.1186/s12891-023-06939-0

**Published:** 2023-10-17

**Authors:** Wu-Gen Li, Rou Zeng, Yong Lu, Wei-Xiang Li, Tong-Tong Wang, Huashan Lin, Yun Peng, Liang-Geng Gong

**Affiliations:** 1 Department of Radiology, the Second Affiliated Hospital of Nanchang University, No. 1Minde Road, Nanchang, Jiangxi 330006 China; 2 Department of Radiology, Xinjian County People’s Hospital, Nanchang, 330103 China; 3 Department of Pharmaceuticals Diagnosis, GE Healthcare, Changsha, Hunan 410000 China

**Keywords:** Machine learning, Occult vertebral fractures, Radiomics, CT

## Abstract

**Purpose:**

To develop and evaluate the performance of radiomics-based computed tomography (CT) combined with machine learning algorithms in detecting occult vertebral fractures (OVFs).

**Materials and methods:**

128 vertebrae including 64 with OVF confirmed by magnetic resonance imaging and 64 corresponding control vertebrae from 57 patients who underwent chest/abdominal CT scans, were included. The CT radiomics features on mid-axial and mid-sagittal plane of each vertebra were extracted. The fractured and normal vertebrae were randomly divided into training set and validation set at a ratio of 8:2. Pearson correlation analyses and least absolute shrinkage and selection operator were used for selecting sagittal and axial features, respectively. Three machine-learning algorithms were used to construct the radiomics models based on the residual features. Receiver operating characteristic (ROC) analysis was used to verify the performance of model.

**Results:**

For mid-axial CT imaging, 6 radiomics parameters were obtained and used for building the models. The logistic regression (LR) algorithm showed the best performance with area under the ROC curves (AUC) of training and validation sets of 0.682 and 0.775. For mid-sagittal CT imaging, 5 parameters were selected, and LR algorithms showed the best performance with AUC of training and validation sets of 0.832 and 0.882. The LR model based on sagittal CT yielded the best performance, with an accuracy of 0.846, sensitivity of 0.846, and specificity of 0.846.

**Conclusion:**

Machine learning based on CT radiomics features allows for the detection of OVFs, especially the LR model based on the radiomics of sagittal imaging, which indicates it is promising to further combine with deep learning to achieve automatic recognition of OVFs to reduce the associated secondary injury.

## Introduction

Occult vertebral fractures (OVFs) are fractures with a small degree of trabecular disruption, where the intact cortical bone overlying the vertebral collapse makes it difficult to directly visualize on X-ray and computed tomography (CT) scans [1]. Based on the Genant semi-quantitative system, which classifies the degree of vertebral collapse into grades 0, 1, 2, 3, and 4, the change in OVF vertebrae is too small to be easily visualized, and is therefore graded = 0 [2]. OVFs may be caused by low- energy trauma in elderly patients suffering from osteoporosis, or may occur in the vertebrae of patients subjected to high-energy trauma and present as chest, abdomen, or back pain [3]. Given the advantages of CT in visualizing organs, soft tissue, and bones, chest/abdomen CT is considered a routine diagnostic modality for evaluating the etiology of chest and/or abdominal pain, especially in patients without a history of trauma [4, 5]. Several studies have reported a high rate of OVF detection on CT, reaching up to 30% [6]. Additionally, 79% of the OVFs observed could potentially collapse and develop into typical non-occult fractures within 3 months [7], which may lead to a variety of complications, such as peripheral soft tissue injury and spinal cord compression [7]. Therefore, it is necessary to identify potential OVFs for early disease management [1].

Magnetic resonance imaging (MRI), particularly fat suppressed sequence such as short tau inversion recovery (STIR) sequence, has become the gold standard for the diagnosing of occult fractures because of its high sensitivity in detecting bone marrow edema associated with occult fracture, which is inconspicuous on conventional CT [8]. MRI, however, is typically not considered the first line of investigation for patients without a history of trauma or any specific symptoms. Additionally, MRI might not be readily available in primary medical institutions especially in developing countries, due to its high cost and the need for specialized staff and facilities. Dual-energy CT scans show good diagnostic performance for assessing traumatic bone marrow damage through the visual and quantitative analysis of material decomposition technology. This method, however, requires specialized equipment and specific technical expertise, limiting its clinical use [9].

Radiomics can transform conventional medical images into mineable high-dimensional data, permitting the extraction of non-visible features, and subsequent analysis and modeling for noninvasive phenotyping and outcome prediction [10]. Radiomics and texture analysis (TA) have been widely used in the musculoskeletal system. Zaworski et al. [11] found that MRI-based TA of trabecular bone could evaluate vertebral bone fragility while discriminating between osteoporosis patients with or without a history of fracture. Sollmann et al. [12] reported that some texture features, based on CT of the vertebral body, showed a strong correlation with finite element analysis results, indicating that radiomics parameters can be used to evaluate the vertebral load. Tabari et al. [13] demonstrated that some texture parameters of vertebral trabeculae on lumbar spine CT in patients with anorexia nervosa were different than those in the healthy control group, suggesting that TA might provide information about bone health. These results indicated that radiomics can help predict poor bone health, although its utility for identifying OVFs remains unknown. Additionally, machine learning algorithms, such as support vector machine (SVM), logistic regression (LR), and Bayes, are suited for discovering predictive patterns from complex and large amounts of data, so they have been widely used in radiomics [14].

We hypothesized that although the changes in the height and shape of vertebrae with OVFs are insufficient to be visualized on CT images alone, subtle bone structure interruptions and bone marrow edema can actually be distinguished using CT radiomics features. The objectives of the present study were: (1) to evaluate the diagnostic performance of vertebral radiomics on axial and sagittal slices from chest/abdomen CT combined with machine learning algorithms to identify vertebrae with OVFs; and (2) to identify the best imaging position and machine learning algorithm for the diagnosis of OVFs using radiomic features.

## Materials and methods

### Study participants

This retrospective study was approved by our institutional review board, the need for written informed consent was waived. We searched our institutional picture archiving and communication system, and identified 314 patients with fresh vertebral fractures between January 2018 and August 2021. Inclusion criteria were as follows. (1)Patient with fresh vertebral fractures with bone marrow edema confirmed by thoracic/lumbar MRI. Bone marrow edema was identified as significantly high signal on STIR and T2WI sequences and low signal on T1WI; (2)with chest/abdominal CT scans within 48 h of the MR examination; (3) Patients with at least one fresh fracture vertebra recognized as OVFs ,that is the fractural vertebral without visual reduction in height (Genant grade = 0) on initial CT imaging [12].

**Fig. 1:**
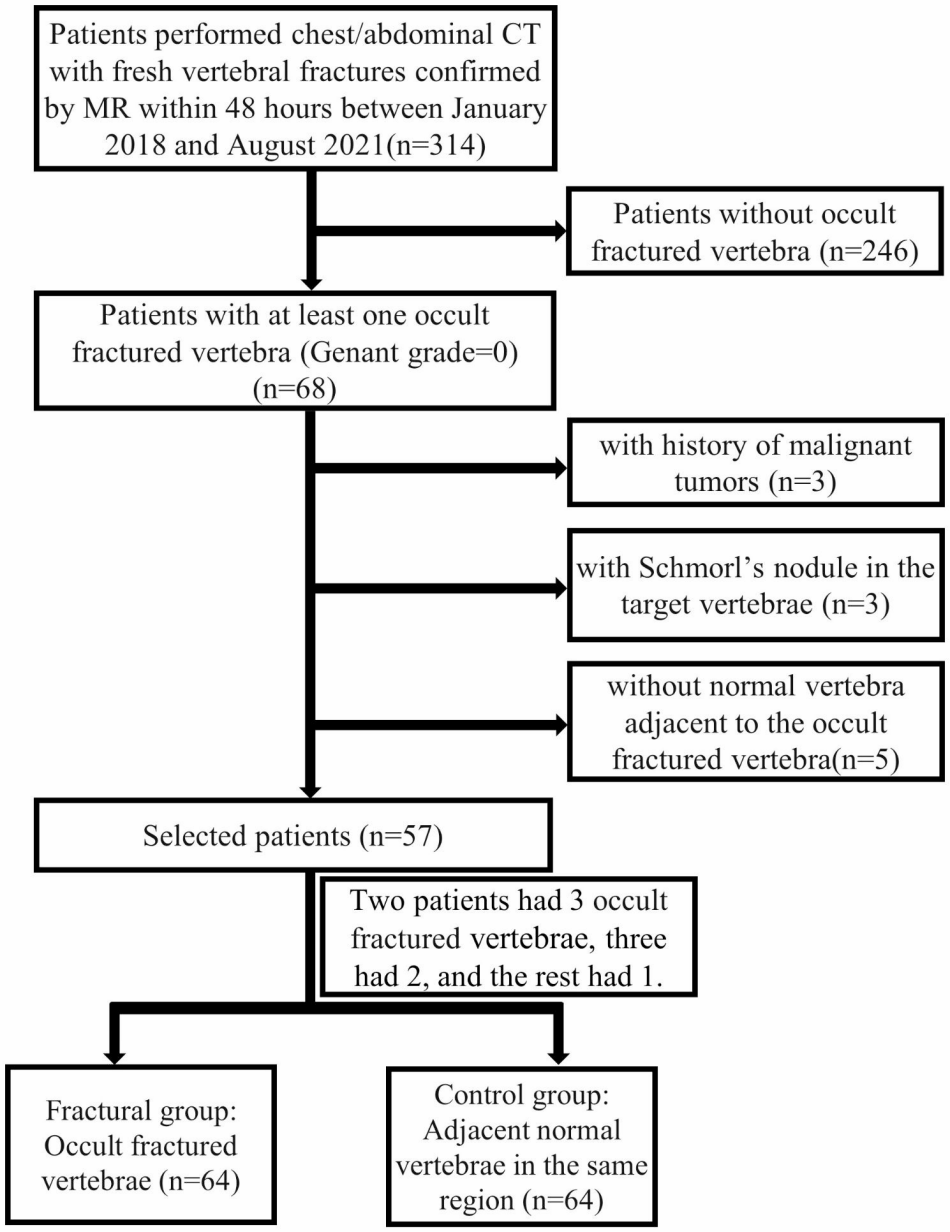
Flow diagram showing study inclusion and exclusion criteria

The spine was divided into 3 regions: thoracic spine (T, Th1–Th10); thoracic-lumbar junction (TLJ, Th11–L1); and lumbar spine (L, L2–L5) [15]. For each OVF vertebra that was included, an adjacent normal vertebra from the same region was selected for the control group. The exclusion criteria were: (1) patient with diffuse metabolic bone disease, malignant tumors, and spinal infections; (2) no normal vertebrae adjacent to the OVF vertebra(e) in the same region; (3) Schmorl’s nodes in the target vertebrae; and (4) significant artefact affecting scan interpretation. 64 OVF vertebrae and 64 corresponding adjacent normal vertebrae in 57 patients deemed eligible for study inclusion (Fig. 1).

### Data acquisition and postprocessing

The images were acquired with Philips Brilliance iCT 256 slice and Brilliance 16 Slice CT scanners. The scanner collimators 16 × 0.75 mm and 256 × 0.625 mm, respectively, peak kilovoltage was 120KV, and the tube current time product was 90–170 mAs. The slice thickness of the original axial images was 1.0 mm, with a matrix of 512 × 512 mm. No intravenous contrast material was administered. For each individual, the thin-slice data was imported into a workstation (IntelliSpace Portal workstation 9.0, Philips Healthcare) for multi-planar reconstruction. Using the axial and sagittal planes as reference, single images from the mid-sagittal and mid-axial planes of the target vertebrae with a slice thickness of 1.0 mm were saved in Digital Imaging and Communications in Medicine (DICOM) format.

The results of the vertebral body evaluation in all CT reports were recorded. The initial reports were written by a junior radiologist after reading the CT images. Then, a senior radiologist reviewed the images again and issued a final report based on the initial one. The absence of a specific description of the vertebral body indicated that the radiologist considered the vertebral body to be normal.

### MRI protocol

MRI examinations of the spine were obtained using a 1.5-T MRI (Signa Hdx; GE Healthcare) with a dedicated spine surface coil. The following sequences were obtained in sagittal orientation: a standard T1-weighted fast spin echo (300 repetition time (TR); 12 echo time (TE); section thickness, 4 mm; gap, 0.4 mm), a T2-weighted turbo spin echo (2120 TR; 102 TE; section thickness, 4 mm; gap, 0.4 mm). and a STIR sequence (2080 TR; 102 TE; section thickness, 4 mm; gap, 0.4 mm).

### Radiomics feature extraction

**Fig. 2:**
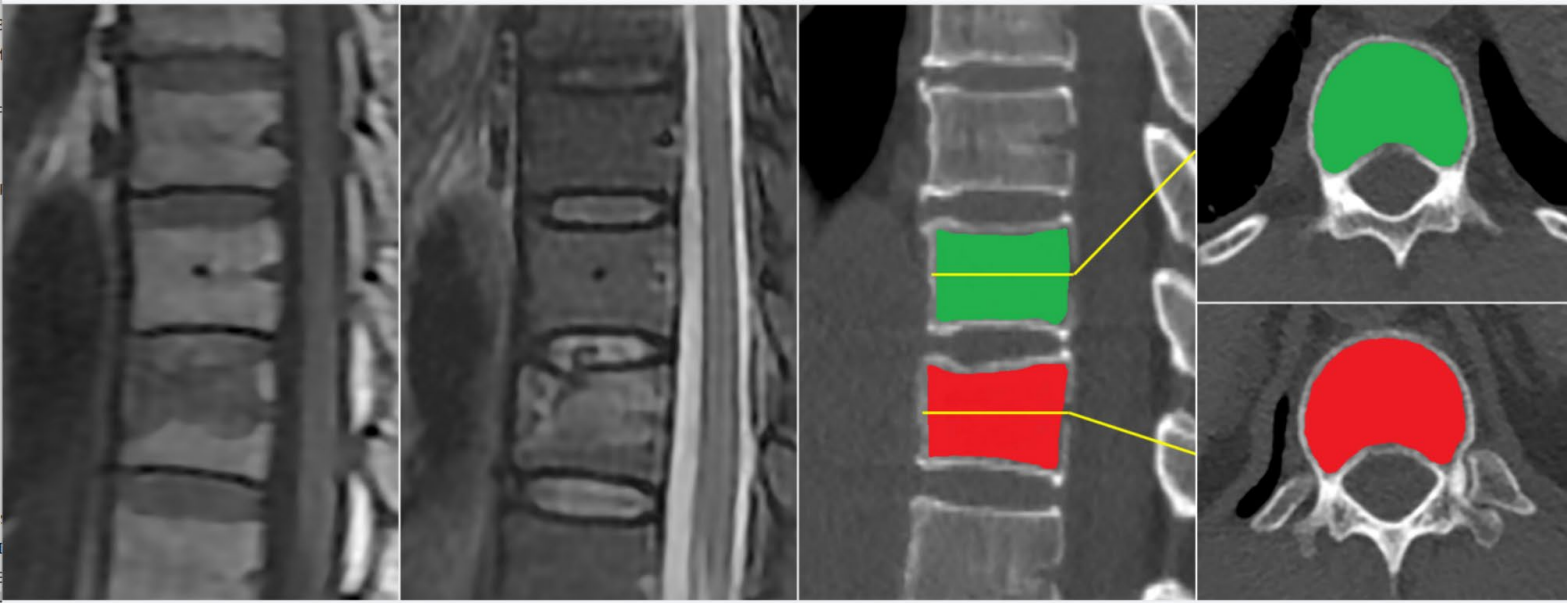
Images of a 48-year-old man with occult vertebral fracture (OVF). (**A**) T1-weighted magnetic resonance (MR) image, and corresponding (**B**) sagittal short tau inversion recovery (STIR) image confirm bone marrow edema without significant vertebral compression at T11, diagnosed as an OVF. T12 was chosen as the control group. (**C**) Free-hand regions of interest (ROIs) drawn on T11 (red) and T12 (green) in the constructed mid-sagittal CT image. ROI delineation was restricted to the trabecular bone. ROIs drawn in the corresponding mid-axial images of T11 (**D**) and T12 (E)

All of the images were resampled to a 1 mm × 1 mm × 1 mm voxel size using the “SimpleITK(version 1.2.4)” package in an integration software based on R Programming and Python Artificial Intelligence software kits (AK, version 3.2.0, GE Healthcare) [16]. Freehand regions of interest (ROIs) were delineated for all 128 vertebrae by a single radiologist with 5 years of specialist experience using ITK-SNAP software (version 3.6.0, www.itksnap.org). The ROIs were manually drawn on a single mid-sagittal and mid-axial slice of each vertebral body (Fig. 2). Then, the CT images, together with the related ROIs, were imported into AK software for radiomics feature extraction.

For each vertebra, 107 radiomic features were extracted from both the mid-axial and mid-sagittal images. The radiomic features can be subdivided into 7 classes, as follows: 18 first-order features; 24 Gy-level co-occurrence matrix (GLCM) features; 14 Gy-level dependence matrix (GLDM) features; 16 Gy-level run length matrix (GLRLM) features; 16 Gy-level size zone matrix (GLSZM) features; 5 neighboring gray tone difference matrix (NGTDM) features; and 14 shape features [17].

To assess the interobserver reproducibility of the radiomics features, ROI delineation was repeated by a second reader with 11 years of specialist experience for reproducibility analysis. Intraclass correlation coefficients (ICCs) for the radiomics features were calculated. Radiomics features with good reproducibility (ICC values ≥ 0.8) were included for further analysis [18].

### Model construction

All vertebrae were randomly divided into a training and a validation set, at a ratio of 8:2. Feature selection and dimension reduction were performed in two steps. First, Pearson’s correlation analysis was performed to remove the redundant features with strong correlations, and the threshold for collinearity was set at R > 0.9. Second, the least absolute shrinkage and selection operator (LASSO) regression model was used to construct the signature of the dataset; LASSO set all regression coefficients toward zero based on the regulation weight λ, with many uncorrelated features having coefficients exactly zero. Tenfold cross validation of the minimum criteria was performed to find the best λ, and finally the value of λ with minimum cross validation error was found.

For classification, machine learning models including support vector machine (SVM), logistic regression (LR), and Bayes algorithms were established, based on the remaining CT features in both the sagittal and axial planes. Model construction was performed with 5-fold cross validation. Receiver operating characteristic (ROC) analysis was performed for the machine learning models, and the diagnostic value was expressed as the area under the ROC curve (AUC). The Delong test was used to compare the AUC between the models. The decision curve analysis (DCA) was used to evaluate the clinical usefulness of the radiomics-clinical model.

### Statistical analysis

The statistical analyses were performed with SPSS (version 19.0) and the “One-key AI” platform (https://www.medai.icu), which is based on Pytorch 1.8.0 [19]. Statistical significance was set at a p-value < 0.05. Continuous variables are presented with descriptive statistics: standard deviation (SD), mean, median, and quartile spacing, where appropriate. Z score normalization was performed on all radiomics features to improve the comparability of the various features.

## Results

### Study population

A total of 128 vertebrae were included in the present study, 64 with OVFs, and 64 adjacent normal bodies (including 16 pairs of vertebrae in the thoracic spine, 43 pairs in the TLJ, and 5 in the lumbar spine) from 57 patients (36 women, 21 men; mean age, 63.51 ± 15.54 years). The clinical characteristics of the training and validation sets were shown in Table 1.

**Table 1:** Clinical Factors and CT Features of the Training and Validation Sets

	Training set (n = 102)			Validation set (n = 26)			P value
	OVF(n = 51)	Control(n = 51)	P value	OVF(n = 13)	Control(n = 13)	P value	0.249
Age, year	63.04 ± 15.23			66.85 ± 13.93			
	63.35 ± 15.41	62.73 ± 15.14	0.836	65.62 ± 13.91	68.08 ± 14.40	0.661	
Gender(M/F)	42/60			6/20			0.089
	22/29	20/31	0.687	2/11	4/9		0.645

OVF, occult vertebral fracture; F, female; M, male

### Feature selection

**Fig. 3:**
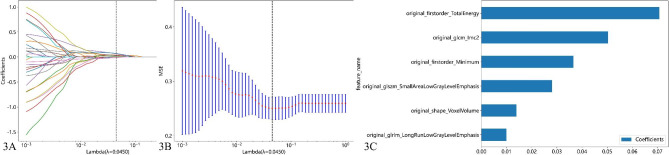
Shows the screening of axial CT radiomics features. (**A**) LASSO coefficient profiles of the features. (**B**) Tuning parameter selection in LASSO; (**C**) The histogram of the coefficients of the selected features

**Fig. 4:**
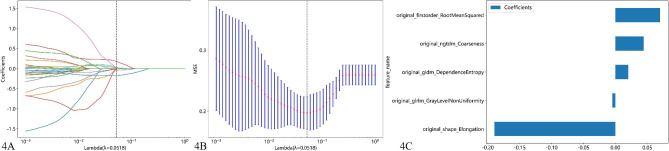
Shows the screening of sagittal CT radiomics features. (**A**) LASSO coefficient profiles of the features. (**B**) Tuning parameter selection in LASSO; (**C**) The histogram of the coefficients of the selected features

Of the 107 radiomic features evaluated, 76 (71.03%) from axial, and 78 (72.90%) from sagittal CT images, showed good reproducibility between the two readers. After selection, a total of 6 features that coefficient value was none zero remained for axial CT imaging. The detail of the features shows in Fig. 3. A total of 5 features that coefficient value was none zero remained for sagittal CT imaging. The detail of the features shows in Fig. 4.

### Model performance

**Fig. 5:**
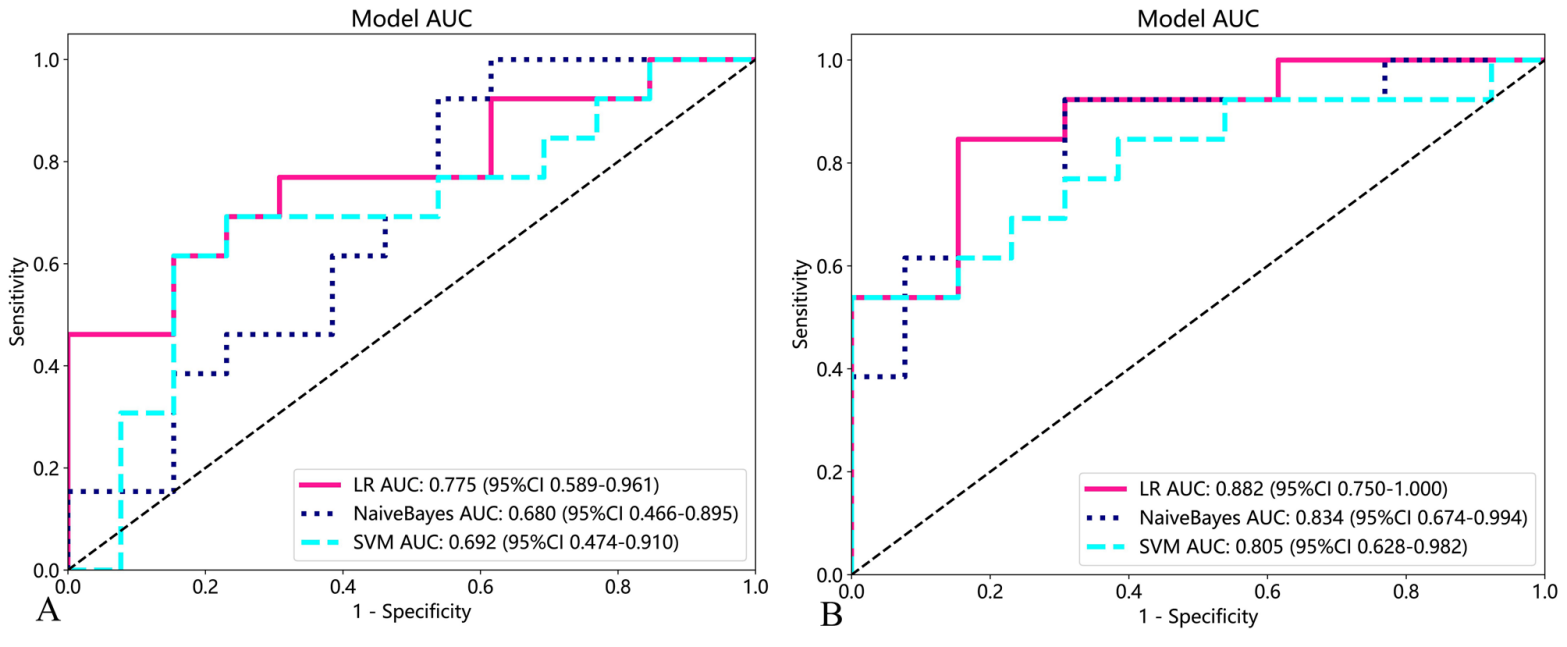
ROC curves of the machine learning model based on axial CT radiomics features (**A**) and sagittal radiomics CT features (**B**) in the validation set. ROC = receiver operating characteristic

**Fig. 6:**
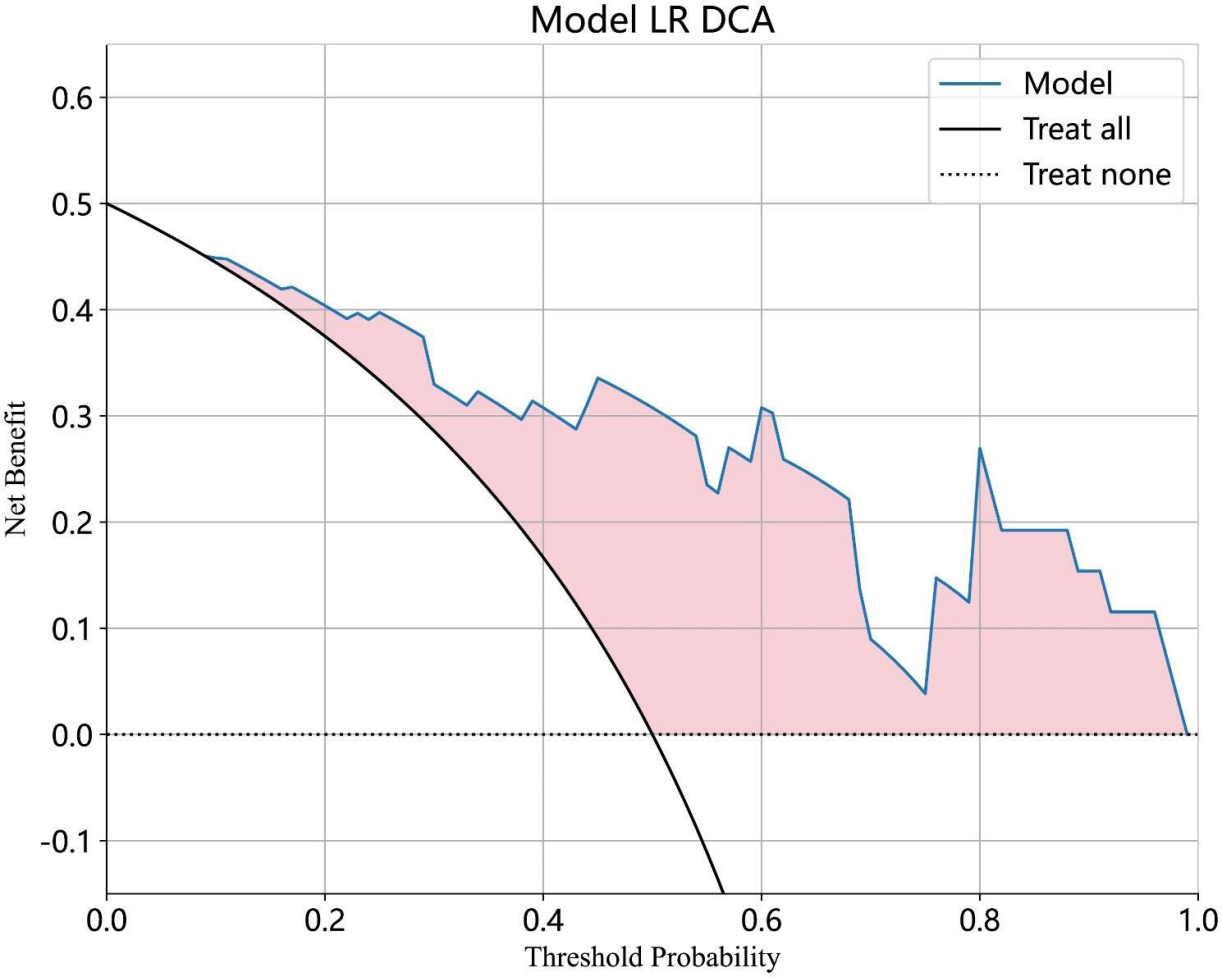
The DCA of LR model based on sagittal CT radiomics features in validation set. DCA = decision curve analysis; LR = logistic regression

The accuracy, sensitivity, and specificity of the models in the axial and sagittal planes are shown in Tables 2 and 3, respectively. The radiomics models based on axial imaging had AUCs of 0.692, 0.775 and 0.680 using the SVM, LR, and Bayes algorithms in the validation set, respectively (Fig. 5A). The radiomics models based on sagittal imaging for the diagnosis of OVFs achieved AUCs of 0.805, 0.882, and 0.834 using the SVM, LR, and Bayes algorithms in the validation set, respectively (Fig. 5B). There was no significant difference in AUCs of different radiomic models (DeLong test, p > 0.05 for each comparison). The LR model based on sagittal CT radiomics yielded the best predictive performance, with an accuracy of 0.846, sensitivity of 0.846, and specificity of 0.846. The DCA demonstrated that the model could provide greater benefit (Fig. 6).

**Table 2:** Diagnostic Performance of Machine Learning Models for Predicting Occult Vertebral Fracture Based on CT Sagittal Imaging

	AUC (95% CI)	ACC	SEN	SPE		AUC (95% CI)	ACC	SEN	SPE
	Training set					Validation set			
SVM	0.893 (0.833, 0.953)	0.814	0.686	0.941		0.805 (0.628, 0.982)	0.769	0.538	1.000
LR	0.837 (0.760, 0.914)	0.804	0.765	0.843		0.882 (0.750, 1.000)	0.846	0.846	0.846
Bayes	0.845 (0.770, 0.919)	0.794	0.824	0.765		0.834 (0.675, 0.994)	0.808	0.923	0.692

SVM, support vector machine; AUC, area under the curve; CI, confidence interval; ACC, accuracy; SEN, sensitivity; SPE, specificity

**Table 3:** Diagnostic Performance of Machine Learning Models for Predicting Occult Vertebral Fracture Based on CT Axial Imaging

	AUC (95% CI)	ACC	SEN	SPE		AUC (95% CI)	ACC	SEN	SPE
	Training set					Validation set			
SVM	0.780 (0.689, 0.870)	0.745	0.863	0.627		0.692 (0.474, 0.910)	0.731	0.615	0.917
LR	0.682 (0.578, 0.786)	0.657	0.431	0.882		0.775 (0.589, 0.961)	0.731	0.462	1.000
Bayes	0.689 (0.586, 0.791)	0.657	0.647	0.667		0.680 (0.466, 0.895)	0.692	0.923	0.462

LR, logistic regression; SVM, support vector machine; AUC, area under the curve; CI, confidence interval; ACC, accuracy; SEN, sensitivity; SPE, specificity

## Discussion

CT is a frequently used diagnostic test for patients presenting with chest and/or back pain in the emergency department [19, 20]. However, the conventional CT, can well display morphologic bone changes, it is unable to detect bone contusions without significant morphologic changes in OVFs [21]. Our study findings show that by selecting and extracting the radiomics features of the vertebral bodies on chest/abdomen CT images, machine learning models combined with CT radiomics achieved a high diagnostic value for OVFs. The diagnostic performance of the radiomics models was higher than that of the radiologist. The CT radiomics model in the sagittal plane showed a higher AUC in differentiating OVF from normal vertebrae than in the axial plane. Among the models developed using SVM, LR and Bayes, the LR-developed model had the best performance.

Previous studies on the use of radiomics in the skeletal system have primarily focused on bone tumors. Chen et al. [22] found that a radiomics nomogram based on MRI could predict early relapse in osteosarcoma. Yin et al. [23] found that radiomics nomograms based on CT and MRI features could be used for the preoperative differentiation of sacral chordomas and giant cell tumors. Several studies have shown promising predictive performances of vertebral CT radiomics for evaluating the integrity and structural changes of the bone. Tabari et al. [13] investigated the utility of trabecular TA, and found that partial radiomic parameters might contribute information about bone health in anorexia nervosa. Muehlematter et al. [24] observed that spine TA combined with SVM allows for the identification of fractural vertebrae. To the best of the authors’ knowledge, the present study is the first to diagnose OVFs using CT radiomics analysis, and the results of our study demonstrated that there were significant differences in some features between the fracture and control groups. These results indicated that although OVFs showed minimal effects on vertebral height, due to the slight dislocation of the cancellous bone and marrow edema, which may be non-visible, the grayscale and distribution changes on CT imaging can be quantified by radiomics features.

Seven classes of features were extracted in the present study. Among them, the first-order feature describes how grey levels within the ROI are distributed. GLCM, GLDM, GLRLM, GLSZM, and NGTDM belong to the texture features designed to evaluate surface textures in 2D images or 3D objects. They are primarily used to represent the gray distribution characteristics of pixels/voxels of discretized gray levels and neighboring pixels/voxels in the same or different directions. The shape features describe the geometric aspects of an ROI [25]. A combination of radiomics features from various levels can better evaluate microstructural changes.

Increased computing power and the successful development of new algorithms have provided several approaches with which to distinguish pathological conditions based on radiomics over the past few decades. In the present study, we built a variety of radiomics models (SVM, LR, and Bayes), based on sagittal and axial planes, for the identification of OVF and normal vertebrae. LR calculates the predicted probability of binary classification by obtaining the weight of each variable through training, and the algorithm is easy to understand. The SVM algorithm is a non-probabilistic classifier defined by a hyperplane that divided the samples into their real classes, with gaps as wide as possible. It is a nonlinear model that may be used for machine learning with small samples [26]. The Bayesian algorithm, a probabilistic classification method, assumes each variable is independent, and calculates its corresponding probability. It then integrates statistics to obtain the final classification probability based on Bayes’ theorem. These three algorithms have advantages not only in small sample data processing and avoiding overfitting, but also in computing power requirements. Their performance has been verified by many studies [26–28]. In this research, the LR model achieved the highest AUC, specificity, and sensitivity, meeting the requirements for the detection of occult fractures in clinical diagnosis and treatment.

Moreover, the AUCs of the models based on the sagittal plane were generally higher than those for the axial plane, which may be related to the method of slice selection during ROI placement in the two planes. The ROIs in the present study were delineated in the mid-sagittal and mid-axial planes, while the bone marrow edema or cancellous bone changes in OVFs always involved partial, instead of whole, vertebral bodies, due to the application of uneven forces. Additionally, bone marrow edema was typically distributed laterally in the vertebrae. The proportion of slices in the mid-axial region involved in bone bruising was small, and even absent, in some cases, while the mid-sagittal region may have included a higher proportion, resulting in a higher range of bone bruising overall in the sagittal than the axial ROIs. Furthermore, although OVFs are more likely to affect the superior endplates, in the present study, the mid-axial, rather than the superior plane, of the vertebral body was selected for evaluation in the early stage of the design because of easier localization, which may facilitate repeatability and subsequent studies. This may also lead to a lower proportion of OVFs involving the mid-axial versus the mid-sagittal slices, resulting in a lower efficacy.

Prior studies have distinguished the obvious vertebral fractures through deep learning. For instance, Yabu et al. employed deep learning to detect fresh vertebral fracture in MRI and yielded favorable efficacy [29]. Kolanu et al. developed a computer-aided design system based on deep learning techniques to identify Genant grades 2/3 vertebral fractures in the routine chest/abdominal CT [30]. Although the purpose of our study is the same as those studies, to find fractures, our study concentrates on OVFs that are difficult to detect with eyes. And they employed the more advanced methodologies. Mohammad et al. combined the deep learning algorithm with radiomics, to achieve lesion classification automatically [18]. We expect combined deep learning-based-automatic segmentation technology with radiomics in future to realize automatic OVF detection.

Several limitations of the present study should be noted. First, the sample size was small, due to the limited time interval between the CT and MRI exams; therefore, additional research with a larger sample size is required to validate the efficacy of the radiomics model. Second, the data were from a single hospital, and the inherent lack of an external validation set limits the conclusions. Third, the distribution of the included vertebrae was not uniform among regions and ages, which may lead to data bias. Most of the selected vertebrae were located in the TLJ region, due to the concentration of stress in this region. The higher proportion of women (61.67%) in this study may be related to the high incidence of osteoporosis caused by the reduction of estrogen after menopause. Finally, although the predictive models showed good performance in this preliminary study, the AUCs of the machine learning based on radiomics in identification of OVFs were mildly lower than that of study using conventional CT features such as attenuation values [31]. It is necessary to increase the sample size, optimize the algorithm, and develop deep learning system to identify OVFs automatically to reduce missed diagnosis in the further studies.

In conclusion, CT radiomics, combined with machine learning, allows for the identification of OVFs not readily appreciable on CT. A LR-developed model based on sagittal CT images demonstrated the best performance out of the three models (SVM, LR and Bayes) examined. This finding shown that the changes of vertebral structure can be quantified and used for diagnosis of OVFs, which indicates it is promising to further combine with deep learning to achieve automatic recognition of OVFs to reduce the associated secondary injury.

## Data Availability

The datasets generated and/or analysed during the current study are not publicly available due [Data security], but are available from the corresponding author on reasonable request.
